# Short Term Isocaloric Ketogenic Diet Modulates NLRP3 Inflammasome *Via* B-hydroxybutyrate and Fibroblast Growth Factor 21

**DOI:** 10.3389/fimmu.2022.843520

**Published:** 2022-04-28

**Authors:** Eun Ran Kim, So Ra Kim, Wonhee Cho, Sang-Guk Lee, Soo Hyun Kim, Jin Hee Kim, Eunhye Choi, Jeong-Ho Kim, Je-Wook Yu, Byung-Wan Lee, Eun Seok Kang, Bong-Soo Cha, Myung-Shik Lee, Jin Won Cho, Justin Y. Jeon, Yong-ho Lee

**Affiliations:** ^1^ Division of Endocrinology and Metabolism, Department of Internal Medicine, Yonsei University College of Medicine, Seoul, South Korea; ^2^ Graduate School, Yonsei University College of Medicine, Seoul, South Korea; ^3^ Department of Hospital Medicine, Yongin Severance Hospital, Yonsei University College of Medicine, Yongin, South Korea; ^4^ Exercise Medicine Center for Diabetes and Cancer Patients, Institute of Convergence Science (ICONS), Yonsei University, Seoul, South Korea; ^5^ Department of Laboratory Medicine, Yonsei University College of Medicine, Seoul, South Korea; ^6^ Brain Korea 21 PLUS Project for Medical Science, Yonsei University College of Medicine, Seoul, South Korea; ^7^ Department of Microbiology and Immunology, Institute for Immunology and Immunological Diseases, Yonsei University College of Medicine, Seoul, South Korea; ^8^ Institute of Endocrine Research, Yonsei University College of Medicine, Seoul, South Korea; ^9^ Severance Biomedical Science Institute, Yonsei Biomedical Research Institute, Yonsei University College of Medicine, Seoul, South Korea; ^10^ Department of Systems Biology, Glycosylation Network Research Center, Yonsei University, Seoul, South Korea

**Keywords:** β-hydroxybutyrate, FGF21, IL-1β (interleukin 1β), Ketogenic diet (KD), isocaloric, NLRP3 inflammasome

## Abstract

A ketogenic diet (KD) is known to have beneficial health effects. Various types of KD interventions have been applied to manage metabolic syndrome based on modification of diet parameters such as duration of intervention, macronutrient components, and total calories. Nevertheless, the beneficial health impact of isocaloric KD is largely unknown, especially in healthy subjects. The present study investigated the acute effects of a 3-day isocaloric KD. In this non-randomized intervention study, we recruited 15 healthy volunteers aged 24-38 years (7 men and 8 women) and placed them on an isocaloric KD restricting intake of carbohydrates but not energy (75% fat, 20% protein, 5% carbohydrate) for 3 days. Biochemical profiles and laboratory measurements were performed. Peripheral blood monocular cells were cultured, and measured cell stimulated cytokines. After short-term isocaloric KD, subjects lost body weight and serum free fatty acid levels were increased. These results accompanied elevated serum β-hydroxybutyrate (BHB) concentration and fibroblast growth factor 21 (FGF21) levels and improved insulin sensitivity. Regarding the direct effect of BHB on inflammasome activation, interleukin-1β (IL-1β) and tumor necrosis factor-α secretion in response to adenosine triphosphate or palmitate stimulation in human macrophages decreased significantly after isocaloric KD. In *ex-vivo* experiments with macrophages, both FGF21 and BHB further reduced IL-1β secretion compared to either BHB or FGF21 alone. The inhibitory effect of FGF21 on IL-1β secretion was blunted with bafilomycin treatment, which blocked autophagy flux. In conclusion, isocaloric KD for 3 days is a promising approach to improve metabolic and inflammatory status.

**Clinical Trial Registration:**

clinicaltrials.gov (NCT02964572).

## Introduction

Obesity and diabetes are global health concerns. A predominant clinical and pathological characteristic of these conditions is chronic low-grade inflammation, a key component in the pathogenesis of insulin resistance and metabolic syndrome (1). Although medical intervention to counteract obesity and diabetes is a common solution, dietary and lifestyle changes are also critical factors in resolving these problems (2). Ketogenic diet (KD) is known to have beneficial health effects. It has been utilized for nearly a century to treat epilepsy. Recent evidence suggests beneficial effects from KD to manage metabolic disorders by reducing body weight and fat accumulation and ameliorating liver steatosis and insulin resistance (2–4). The KD regimen has been shown to improve cardiomyopathy in a diabetic mouse model (db/db) and in humans by unloading mitochondrial burden (5, 6). Patients with obesity and type 2 diabetes mellitus gain better control of glucose levels when on a very low carbohydrate diet (7, 8). In addition, dietary effects were found to improve and prevent cognitive decline in human trials of dementia (9).

A KD is comprised of very low carbohydrate content (5%-10% of total daily calorie intake, or 20-50 g/day), which can induce ketosis *via* production of β-hydroxybutyrate (BHB), acetoacetate (AcAC), and acetone, mainly in the liver (10). Hepatocyte-derived AcAc in macrophages suppressed hepatic stellate cells, resulting in protection against tissue fibrosis (11). BHB has been exclusively studied among other ketone bodies. High levels of BHB ameliorate inflammation and increase lean mass in patients with multiple sclerosis (12). Lipopolysaccharide (LPS)-induced NLRP3 inflammasome activation and IL-1β production are suppressed in macrophages that protect against muscle loss, suggesting a possible therapeutic target to fight pathophysiology associated with inflammation (13). BHB also acts on neutrophils, which inhibit NLRP3 inflammasome activation and block IL-1β secretion in mice and humans (14, 15). Further, it acts as a signal molecule by binding to hydroxycarboxylic acid receptor 2 (16, 17). There was a report that fibroblast growth factor 21 (FGF 21) increases insulin sensitivity (18) and the BHB signaling pathway (19). However, the precise mechanisms underlying these effects remain incompletely understood.

Various types of KD interventions have been applied to manage metabolic disorders depending on modification of diet parameters, such as duration of intervention, macronutrient components, and total calorie. The beneficial health impact of isocaloric KD is largely unknown, especially in healthy subjects. Thus, the underlying mechanisms that explain the effect of isocaloric KD are unclear.

Therefore, the present study aimed to investigate the acute effects of isocaloric KD on metabolic parameters in healthy subjects. Furthermore, we evaluated macrophages as mediators of isocaloric KD effects.

## Methods

### Study Design: Isocaloric Ketogenic Diet in Healthy Subjects

**Figure 1:**
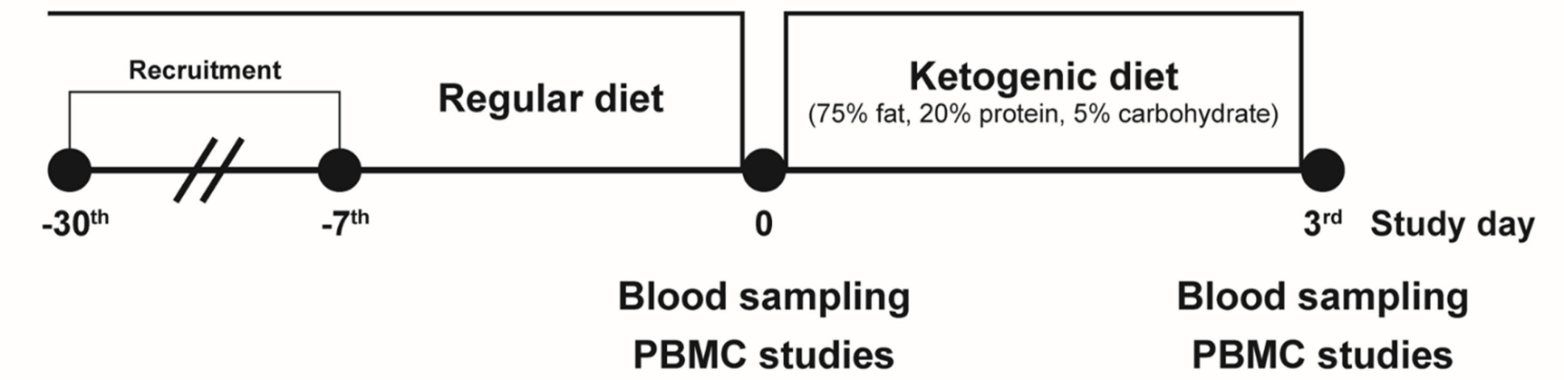
Schematic of the isocaloric ketogenic diet protocol. Healthy volunteers were recruited, and blood samples were collected on day 0 and day 3 of isocaloric ketogenic diet. PBMC, peripheral blood mononuclear cell.

This study was carried out at Severance Hospital. Eligible participants were healthy adults (19-44 years of age) with a BMI of at least 18 kg/m^2^. Exclusion criteria included: any disease including diabetes, hypertension, and dyslipidemia; any current medication; or pregnant women. We recruited 15 healthy volunteers aged 24-38 years of age (7 men and 8 women) and placed them on an isocaloric KD restricting intake of carbohydrates but not energy (75% fat, 20% protein, 5% carbohydrate) for 3 days (**Figure 1**). Total energy (calories)/day were determined by the ‘Dietary Reference Intakes for Koreans’ (20): men, 10.88 MJ (2,600 kcal)/day for age under 30 and 10 MJ (2,400 kcal)/day for age 30 or older; women, 8.79 MJ (2,100 kcal)/day for age under 30 and 7.95 MJ (1,900 kcal)/day for age 30 or older. Participants’ body composition was analyzed by bioelectrical impedance (InBody 720, BioSpace Co., Seoul, Korea) before and after the KD intervention at the same time of day (early morning). This study was approved by the Institutional Review Board at Severance Hospital (4-2016-0795), is registered at clinicaltrials.gov (NCT02964572), and complied with the revised 2008 Declaration of Helsinki. All participants provided written informed consent prior to initiation of any study procedure.

### Outcome Measures

The primary endpoint was change in secretion levels of IL-1β from supernatants of macrophages from baseline to end of the isocaloric KD regimen. Secondary endpoints included: change in secretion levels of tumor necrosis factor-α (TNF-α) from supernatants of macrophages; serum levels of IL-1β, BHB, insulin, FGF21, and other biochemical profiles [free fatty acid (FFA), glucose, and total cholesterol]; and change in body weight from baseline to end of the isocaloric KD regimen.

### Laboratory Measurements

After overnight fasting, serum BHB was determined by an enzymatic assay using a commercial reagent from Nittobo Medical Co., LTD (Tokyo, Japan) and the Hitachi 7600 analyzer. Serum glucose, FFA, and total cholesterol were measured using a Hitachi 7600 automated chemistry analyzer (Hitachi High-Technologies Corporation, Tokyo, Japan). Fasting serum insulin was measured by electrochemiluminescence immunoassay using a Cobas e601 analyzer (Roche Diagnostics, GmbH, Germany). Insulin sensitivity was assessed using the following indices (21): homeostatic model assessment of insulin resistance (HOMA-IR) = [(fasting serum insulin [µU/mL] × fasting serum glucose [mmol/L])/22.5]; and quantitative insulin sensitivity check index (QUICKI) = [1/(log(fasting serum glucose [mg/dL]) + log(fasting serum insulin [µU/mL]))] (22). Serum IL-1β and FGF21 were measured with ELISA using human Quantikine HS ELISA kits (R&D Systems, MN, USA). Assay sensitivities (the minimum detectable levels) for serum IL-1β and FGF21 were 0.033 pg/mL and 8.69 pg/mL, respectively, determined by zero standard +2 standard deviation.

### Isolation and Culture of Peripheral Blood Mononuclear Cells

The differentiation of macrophages from peripheral blood mononuclear cells (PBMCs) and cytokine assays were performed as described in our previous paper (23). Samples of whole blood were collected into acid citrate dextrose tubes. PBMCs were isolated from blood by density centrifugation (20 min at 1,600 × g (without brakes) at 18-20°C) using Ficoll Medium (Ficoll-Paque PLUS, GE Healthcare Life Science, Uppsala, Sweden). After removing the top layer of clear plasma, the PBMC-containing layer was aspirated, and the cells were washed with Dulbecco’s phosphate-buffered saline. Then the cells were re-suspended in RPMI-1640 supplemented with 1% penicillin, 1% streptomycin, and 10% fetal bovine serum. To generate human macrophages, cells were incubated at 1 × 10^6^ cells per ml in 24-well plates in RPMI medium plus 10% fetal bovine serum for 2 hours and then incubated with 20 ng/mL M-CSF for 3 days. After 3 days, the cells were then incubated with fresh RPMI medium plus 10% fetal bovine serum containing 10 ng/mL M-CSF and the medium was freshly replaced every 2 days (total 7 days) as previously described (23).

### Cell Stimulation and Cytokine Assays

Human macrophages were incubated at 1 × 10^6^ cells per ml in 24-well plates in RPMI medium plus 10% fetal bovine serum with 0.1 μg/mL LPS (Sigma-Aldrich, St. Louis, MO, USA, L6529) for 4 hours (24). To stimulate the release of IL-1β, 2 mM adenosine triphosphate (ATP) (Sigma-Aldrich) or 0.2 mM palmitate (Sigma-Aldrich, P9767) was added for the last 1 or 12 hours of incubation, respectively. In addition, to evaluate the direct inhibitory or stimulating effects on NLRP3 inflammasome activation, BHB (1 mM), FGF21 (15, 30 or 100 nM), or bafilomycin (10 nM) were pre-treated for 5 hours before adding LPS and ATP. Supernatants were collected, centrifuged to remove cells and debris, and stored at -80°C for later analysis. IL-1β and TNF-α was measured using ELISA (eBioscience, Waltham, MA, USA, human 88-7261-88 and human 88-7346-88, respectively). Results were normalized to cell number, as determined by the CyQuant cell proliferation assay (Invitrogen, Waltham, MA, USA). Experiments on the participants were repeated up to three times per sample. The examiner conducting these experiments remained blinded to the subject’s status throughout the study.

### Statistical Analysis

All statistical analyses were performed using Prism 8.3.0 (GraphPad Software, San Diego, CA, USA). A normality test was performed for all continuous variables. The effects of isocaloric KD on metabolic parameters and secretion of cytokines from macrophages and the effects of BHB, FGF21, and bafilomycin treatment on secretion of cytokines from macrophages were assessed by two-sided paired t-test or Wilcoxon signed rank test. One-way ANOVA using Tukey’s test or a two-tailed Student’s t-test with the Bonferroni method for adjusting *P*-values for the number of comparisons being made were used to examine differences between treatments in *ex-vivo* experiments. All *P*-values <0.05 were considered statistically significant.

## Results

### Short-Term Isocaloric Ketogenic Diet Changes Metabolic Parameters

**Figure 2:**
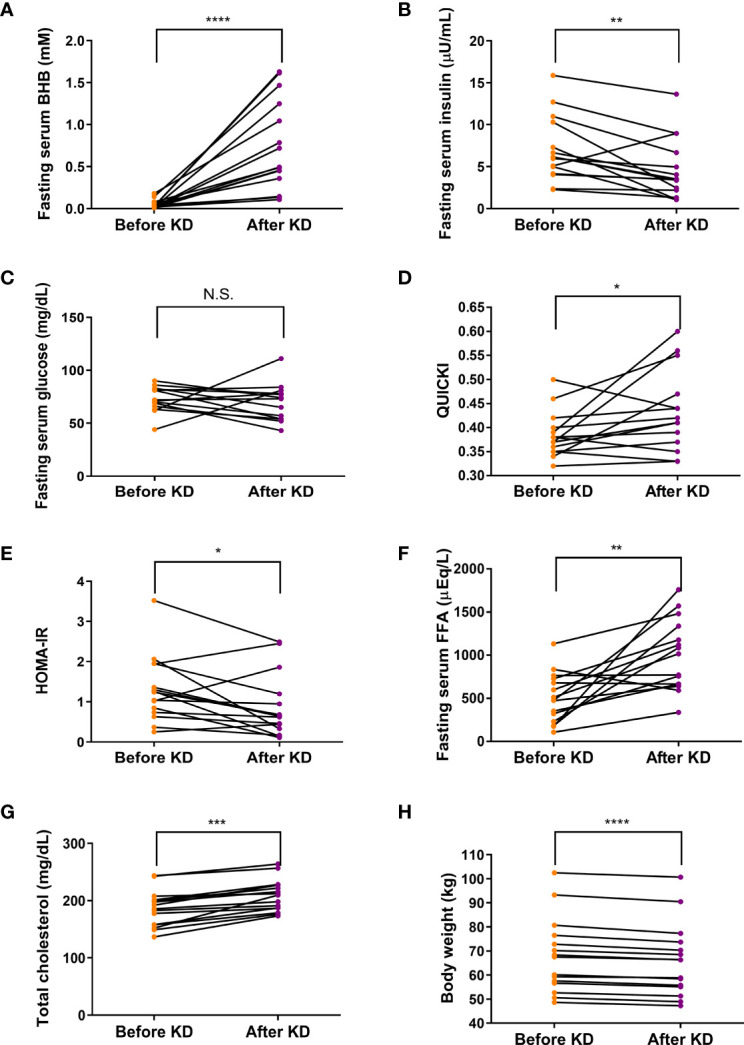
Effects of isocaloric ketogenic diet on metabolic parameters. Fasting serum levels of BHB **(A)**, insulin **(B)**, glucose **(C)**, QUICK **(D)**, HOMA-IR **(E)**, FFA **(F)**, total cholesterol **(G)** from baseline to day 3 of isocaloric KD **(H)**; Body weight changes from baseline to day 3 of isocaloric KD. Two-sided paired t-test or Wilcoxon signed rank test; *P <0.05, **P <0.01, ***P <0.001, and ****P <0.0001 vs. baseline. BHB, β-hydroxybutyrate; FFA, free fatty acid; KD, ketogenic diet; QUICKI, quantitative insulin sensitivity check index; HOMA-IR, homeostatic model assessment of insulin resistance; N.S., non-significant.

After a short-term KD, fasting serum BHB concentration was significantly elevated (median [interquartile range]: 0.03 [0.02 - 0.06] to 0.49 [0.36 - 1.25] mM, *P <*0.0001) (**Figure 2A**). These results accompanied decrease in fasting serum insulin levels (7.00 ± 3.87 to 4.61 ± 3.54 µU/mL, *P* < 0.01) (**Figure 2B**). Interestingly, the isocaloric KD produced no change in fasting serum glucose levels (**Figures 2C**). Thus, isocaloric KD significantly increases insulin sensitivity reflected by improvement in QUICKI and HOMA-IR (**Figures 2D, E**), indicating improved glucose metabolism (25, 26). Fasting serum levels of FFA and total cholesterol were increased after a 3-day isocaloric KD (504.6 ± 288.3 to 998.4 ± 411.1 μEq/L, *P* = 0.002; and 185.6 ± 31.8 to 208.4 ± 27.9 mg/dL, *P <*0.001, respectively) (**Figures 2F, G**). Subjects lost body weight (mean ± standard deviation: -1.87 ± 0.74 kg, *P <*0.0001, **Figure 2H**).

### Short-Term Isocaloric Ketogenic Diet Suppresses Inflammasome Activation

**Figure 3:**
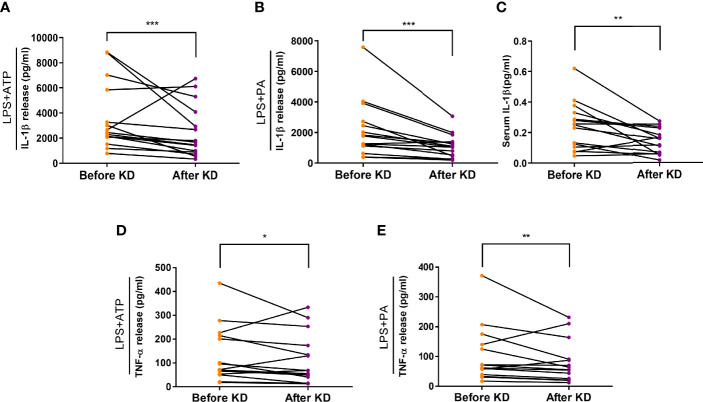
Effects of isocaloric ketogenic diet on secretion of IL-1β and TNF-α from macrophages. Secretion of IL-1β: ELISA assay measurement of IL-1β secretion from macrophages exposed to 2 mM ATP **(A)** or 0.2 mM palmitate **(B)** with 0.1 µg/ml LPS priming; changes in serum IL-1β levels **(C)**; TNF-α secretion from macrophages exposed to ATP **(D)** or palmitate **(E)**. Experiments with macrophage were repeated two or three times per sample; graphs are drawn using mean values of those results per sample, whereas statistical significance is derived from raw data. Two-sided paired t-test or Wilcoxon signed rank test; *P <0.05, **P <0.01, and ***P <0.001 vs. baseline. PA, palmitate; ATP, adenosine triphosphate; LPS, lipopolysaccharide; IL-1β, interleukin-1β; KD, ketogenic diet; TNF-α, tumor necrosis factor-α.

To evaluate the effects of short-term ketogenic diet on inflammasome activation, a primary endpoint of our study, we compared and observed that IL-1β secretion in response to ATP or palmitate stimulation in human macrophages decreased significantly after KD (2,532 [1,882 - 5,645] to 1,719 [840 - 3,883] pg/mL, P <0.001; and 1,637 [1,047 - 2,659] to 1,045 [470 - 1,441] pg/mL, P <0.001, respectively) (**Figures 3A, B**). Furthermore, serum IL-1β was measured by ELISA and was found to have decreased significantly after the isocaloric KD regimen (0.2407 ± 0.0297 to 0.1423 ± 0.019 pg/mL, P <0.01) (**Figure 3C**). In terms of secondary endpoint, secretion of TNF-α in response to ATP or palmitate stimulation also decreased significantly after isocaloric KD (76 [57 - 212] to 64 [43 - 160] pg/mL, P = 0.02; and 65 [38 - 139] to 58 [24 - 93] pg/mL, P = 0.004, respectively) (**Figures 3D, E**).

### Short-Term Isocaloric Ketogenic Diet Suppresses Inflammasome Activation Mediated by FGF21

**Figure 4:**
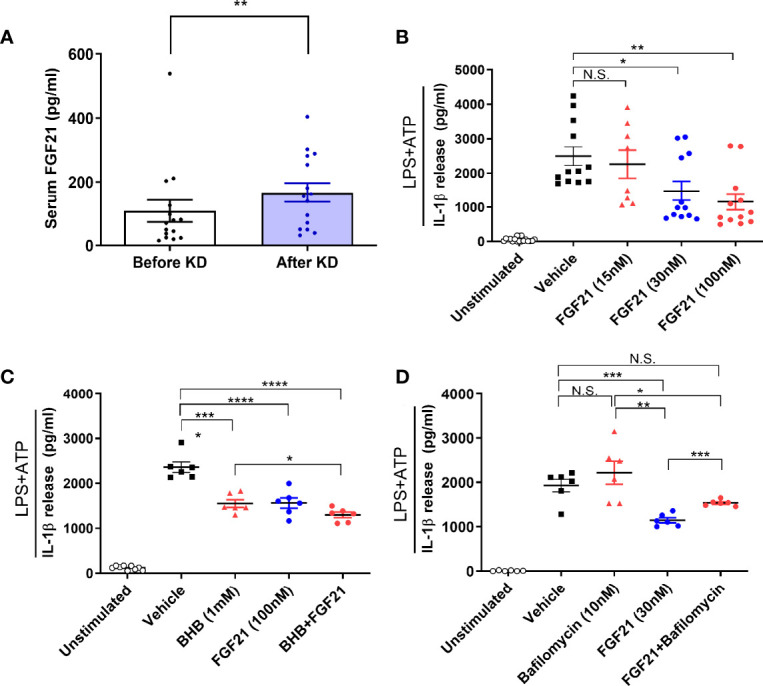
Effects of FGF21 as a mediator of ketogenic diet on NLRP3 inflammasome signaling activation in human macrophages. Changes in serum FGF21 levels from baseline to day 3 of isocaloric KD **(A)**; ELISA assay measurement of IL-1β secretion from macrophages primed with 0.1 µg/ml LPS and stimulated with 2 mM ATP in the presence of various dose of FGF21 (15, 30, 100 nM) for 24h **(B)**, BHB (1 mM), FGF21 (100 nM), or both **(C)**, bafilomycin (10 nM), FGF21 (30 nM), or both **(D)**. Data are represented as mean ± SEM. One-way analysis of variance (ANOVA) and two-sided paired t-test or Wilcoxon signed rank test; *P <0.05, **P <0.01, ***P <0.001, and ****P <0.0001. ATP, adenosine triphosphate; LPS, lipopolysaccharide; FGF21, fibroblast growth factor 21; N.S., non-significant; KD, ketogenic diet; IL-1β, interleukin-1β; BHB, β-hydroxybutyrate.

To investigate whether FGF21 mediates the KD effect on ameliorating inflammasome activation, we measured serum FGF21 levels and found it increased after KD (mean ± standard error of the mean, 108.25 ± 34.1 to 167.28 ± 28.47 pg/mL, *P <*0.01) (**Figure 4A**). To further assess the direct effect of FGF21 on human macrophages, we treated FGF21 prior to assessing inflammasome activation with LPS and ATP. IL-1β secretion was significantly reduced in a dose dependent manner (**Figure 4B**). Both FGF21and BHB further reduced IL-1β secretion levels compared to either BHB or FGF21 alone (**Figure 4C**). In addition, FGF21 is known to augment autophagy in islets (27). We further tested if higher FGF21 levels with isocaloric KD was also related to autophagy activation and found that the inhibitory effect of FGF21 on IL-1β secretion was blunted with bafilomycin treatment, which blocked autophagy flux (**Figure 4D**).

## Discussion

**Figure 5:**
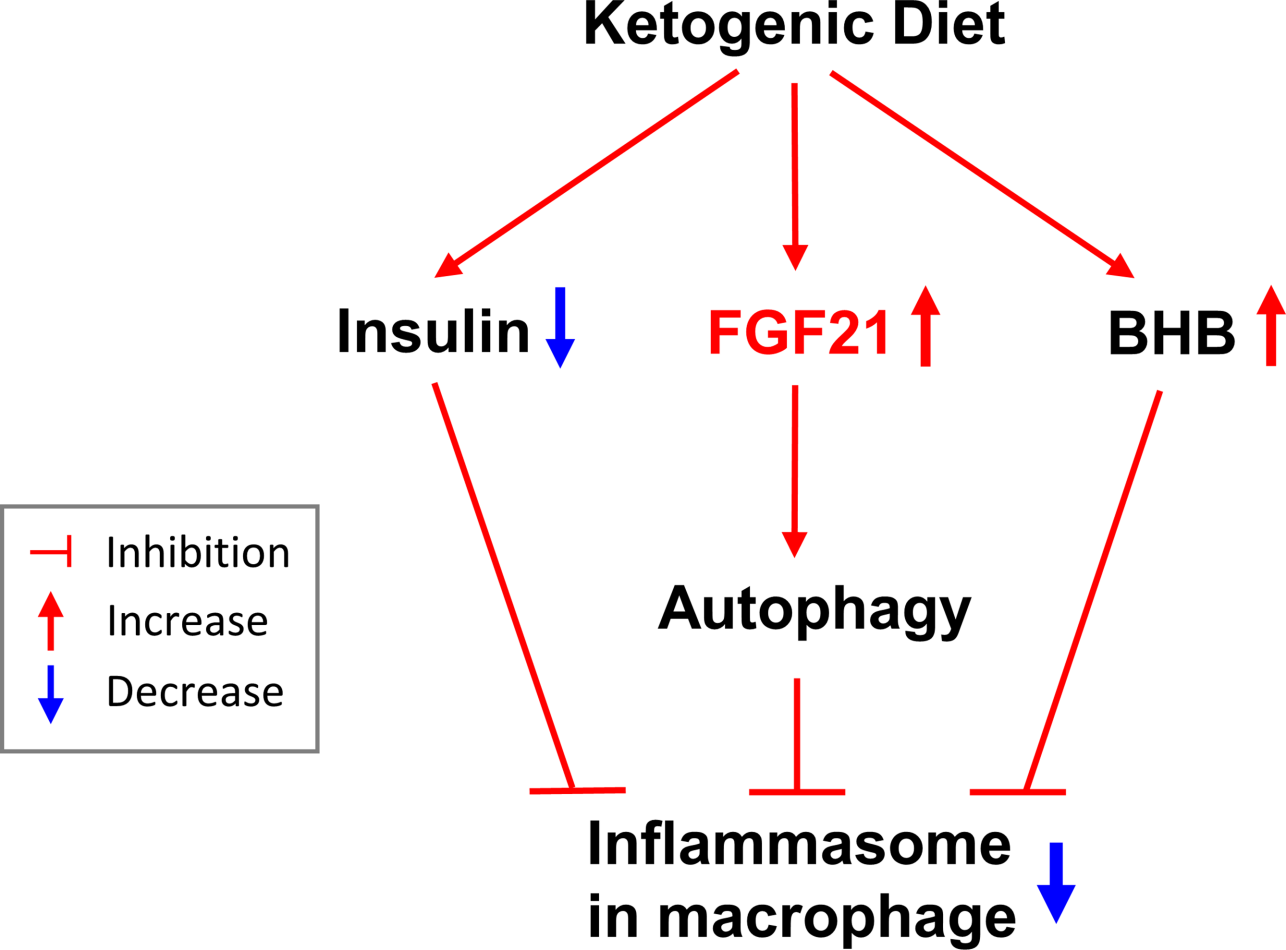
The summary of effects of the isocaloric ketogenic diet.

In this short-term isocaloric KD study, we showed that insulin sensitivity improved with low serum insulin levels, although there was no difference in blood glucose levels. The regimen reduced body weight and increased mobilization of body fat in healthy subjects. High BHB and low IL-1β levels were observed after isocaloric KD. Reduced IL-1β levels were also detected along with increased FGF21 levels in serum. In a test of the direct effect of FGF21 on macrophages, FGF21 was seen to reduce macrophage IL-1β secretion, and its inhibitory effect was blunted by an autophagy inhibitor (**Figure 5**).

It is important to note that there were no changes in glucose levels but insulin levels were low, suggesting that KD improved insulin sensitivity in a short period of time. BHB is involved in the regulation of glucose homeostasis. After 21 days of KD in diabetes patients, glucose and insulin levels significantly were reduced with high BHB levels in serum (28). A very low carbohydrate diet for 8 weeks in men and women with obesity improved HOMA-IR (29).

A recognized effect of KD is reduction in body weight (30, 31), which may explain most of its metabolic benefits. However, most studies were of long duration and investigated prolonged impact, making it difficult to differentiate between KD per se and weight reducing effect. We demonstrated that, even in a short period of time without reducing calorie intake or a large decrement in body weight, isocaloric KD can mobilize fat and change inflammasome activity in immune cells, a potential advantage for improving metabolic regulation (32).

Previously, Vandanmagsar et al. reported that NLRP3 and IL-1β are associated with insulin resistance in mice with obesity (33). In that study, insulin sensitivity improved in mice that were NLRP3 deficient. BHB deactivates NLRP3 and resolves inflammatory diseases (14, 15). In our study, inflammasome activation was significantly suppressed in ATP/LPS-induced human macrophages with isocaloric KD as well as with direct treatment of BHB. In line with these findings, we have previously reported that SGLT2 inhibitor, a glucose lowering drug, regulates NLRP3 inflammasome in macrophages by increasing serum BHB levels and lowering insulin levels in patients with type 2 diabetes mellitus (23).

Along with the high levels of BHB after isocaloric KD, we also detected an increase in serum FGF21. FGF21 is a hormone widely expressed in multiple organs. It was first identified in the liver (34, 35). Hepatic FGF21 mRNA expression levels were induced in mice that were fed a KD for 30 days (36). Serum FGF21 levels increased up to four-fold with prolonged fasting in healthy subjects (25, 37). Exogenous FGF21 has been shown to reduce body weight gain and fat content in diet-induced obese (DIO) or ob/ob genetically obese mouse models. Insulin levels were significantly reduced with FGF21 treatment in a dose dependent manner in the DIO mouse model, indicating an improvement in insulin sensitivity (38). In addition, FGF21 controls triglyceride levels in rodents (39) and in humans (40).

In type 2 diabetes mellitus, NLRP-IL-1β Inflammasome is a key factor in the development of insulin resistance (41). Since FGF21 agonists suppress the inflammasome after reduction of sequential inflammatory processes (42, 43), reduced serum IL-1β levels may be affected by not only BHB activity but also by FGF21 activity. Among human FGF21 receptors, FGF21 receptor 1 and β-klotho are expressed in multiple tissues, including PBMCs (https://www.proteinatlas.org/ENSG00000134962-KLB/blood, https://www.proteinatlas.org/ENSG00000077782-FGFR1/blood). After direct treatment of FGF21 on human macrophages, we observed reduced cytokine secretion related to the inflammasome. When patients with obesity and type 2 diabetes mellitus lose body weight, FGF21 levels also decrease (44). This may be due to improvement in FGF21 signaling sensitivity similar to the effect on insulin resistance, which is ameliorated by loss of body weight. Taken together, our findings suggest that isocaloric KD not only increases FGF21 levels but also improves FGF21 signaling pathways thereby lowering inflammatory activation.

Consistent with these findings, a recent study reported that serum FGF21 levels can predict NAFLD improvement in patients with a very low caloric KD (32); fatty liver is also improved with increased tissue FGF21 mRNA expression levels in intermittent KD [abstract: diabetes 2020 Jun; 69 (Supplement 1):1957-P]. FGF21 is also known to reduce hepatic glucose production (45); however, we did not observe any changes in glucose levels. This may possibly be due to the acute response levels of FGF21 to the isocaloric KD regimen. FGF21 global knockout mice on a KD had opposite phenotypes, such as body weight gain, insulin resistance, and inflammatory state. In addition to the role of FGF21 in glucose and insulin regulation, it has a significant impact on fat mobilization and oxidation (46, 47). In our study, 3 days of isocaloric KD was sufficient to induce an increase in FGF21 levels and a change in FFA levels in healthy subjects. In mice, a 24-hour fasting period was sufficient to increase FGF21 levels (36, 48). Increased FGF21 levels with a KD also mimics the effects of exercise (49). Therefore, our study suggests that isocaloric KD has positive effects in healthy subjects, even in the short term.

Multiple studies have explained that FGF21 augments and exerts its effects through autophagy. For example, fasting induced exogenous FGF21 significantly improved obese phenotypes of mice with increased autophagy-related proteins and autophagosomes (27, 50–52). However, the role of autophagy in isocaloric KD, especially in macrophages of healthy individuals, has not been thoroughly studied. The current study showed that the suppressive effect of FGF21 on inflammasome activation in human macrophages was at least partially mediated through the autophagy signaling pathway. Since autophagy controls oxidative mediated inflammation and rescues macrophages from inflammatory processes (53), the effect of the isocaloric KD–FGF21–autophagy axis on macrophages provides a clear health benefit.

Our results suggest that low caloric intake or fasting was not necessary to induce ketosis. Isocaloric KD for 3 days is sufficient to mobilize fat depot with incremental rise in BHB levels and to improve glucose metabolism by increasing insulin sensitivity. This diet regimen suppresses inflammasome activity in macrophages by increasing FGF21 levels and inducing autophagy, resulting in promising health benefits related to improved metabolic and inflammatory status.

## Data Availability Statement

The raw data supporting the conclusions of this article will be made available by the authors, without undue reservation.

## Ethics Statement

The studies involving human participants were reviewed and approved by Institutional Review Board at Severance Hospital (4-2016-0795). The patients/participants provided their written informed consent to participate in this study.

## Author Contributions

EK, SK and S-GL wrote the manuscript, analyzed data, and performed the statistical analysis; SHK, JK, EC, and WC conducted the experiments and contributed to acquisition of data; JJ, J-HK, B-WL, ESK, B-SC, M-SL, J-WY, and JC provided critical review, advice, and consultation throughout. Y-HL contributed to the conception and design of the study, the interpretation of data, and the drafting of the manuscript. Y-HL is the guarantor of this work and, as such, had full access to all the data in the study and takes responsibility for the integrity of the data and the accuracy of the data analysis. All authors contributed to the article and approved the submitted version.

## Funding

This work was supported by a National Research Foundation of Korea (NRF) grant funded by the Korean Government (MSIP) [NRF-2016R1A5A1010764] and [NRF-2019R1l1A1A01063695] and was also supported by a faculty research grant of Yonsei University College of Medicine for (6-2015-0069). The study funders were not involved in the design of the study; the collection, analysis, and interpretation of data; writing the report; and did not impose any restrictions regarding the publication of the report.

## Conflict of Interest

The authors declare that the research was conducted in the absence of any commercial or financial relationships that could be construed as a potential conflict of interest.

## Publisher’s Note

All claims expressed in this article are solely those of the authors and do not necessarily represent those of their affiliated organizations, or those of the publisher, the editors and the reviewers. Any product that may be evaluated in this article, or claim that may be made by its manufacturer, is not guaranteed or endorsed by the publisher.

## Abbreviations

AcAC, acetoacetate; ATP, adenosine triphosphate; BHB, β-hydroxybutyrate; DIO, diet-induced obese; FGF21, fibroblast growth factor 21; FFA, free fatty acid; HOMA-IR, homeostatic model assessment of insulin resistance; IL-1β, interleukin-1β; KD, ketogenic diet; LPS, lipopolysaccharide; N.S., non-significant; PA, palmitate; PBMC, peripheral blood mononuclear cell; QUICKI, quantitative insulin sensitivity check index; TNF-α, tumor necrosis factor-α.
